# Supplementary material for: Probing for intentions: The early readiness potential does not reflect awareness of motor preparation
Source: Imaging Neurosci (Camb). 2025 Feb 7;3:imag_a_00465. doi: 10.1162/imag_a_00465 (PMC12319783; doi:10.1162/imag_a_00465)
Supplement: Supplementary Material [file imag_a_00465-supp.pdf]

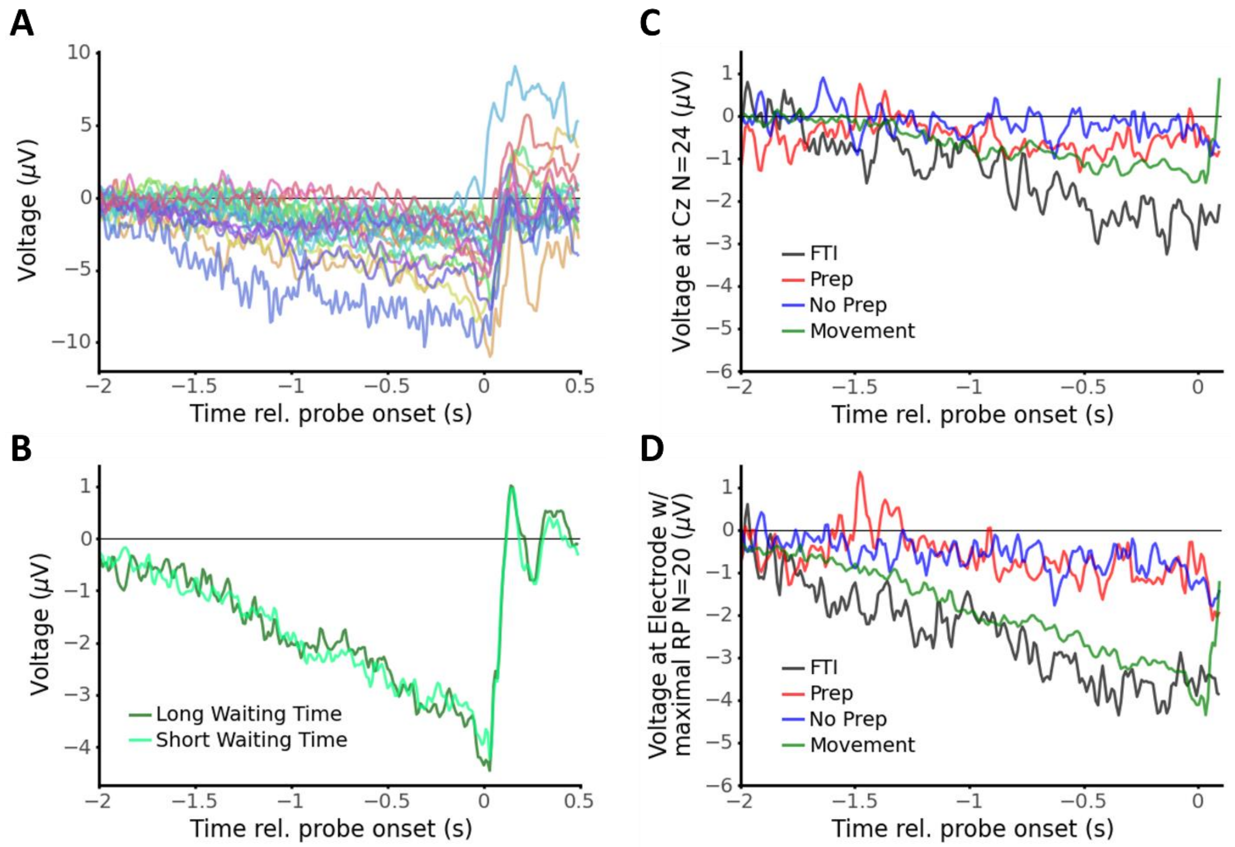

Supplementary Figure 1: Supplementary ERP findings. **A:** Individual participants' readiness potentials in Movement trials after selecting the electrode with maximal negativity (Cz n=9, FCz n=9, Fz n=2). **B:** Unlike Schurger (2018), we did not find a noticeable difference in RP amplitude for trials with relatively short or long waiting-times (by-participant median split after removing waiting times less than 2.5 seconds). Longer waiting times were more likely to be pre-empted by a probe, potentially leading to this lack of difference. **C:** Grand-averaged ERPs (averaged across trials, and then averaged across participants) for all 24 participants at electrode Cz. Lower RP amplitude emerges due to inclusion of participants that did not show an RP and selecting Cz instead of electrodes at which RP is maximal. **D:** Grand-averaged RP (averaged across trials, then averaged across participants using our selection criteria as in the main article) using traditional averaging methods instead of mixed-effects modeling. Findings are consistent with mixed-effects models.

Inclusion criteria	Condition	Slope [-1s, 0s]	95% CI	Amplitude [-0.25s ,0s]	95% CI	BF_{10} vs Prep	BF_{10} vs No Prep.	BF_{10} vs FTI
Visible RP (n=20/24, main text)	Prep.	-0.509 mV/s	(-0.904, -0.115)	-1.102 mV	(-2.671, 0.466)	N/A	**0.091**	1.584
	No Prep.	-0.661 mV/s	(-0.939, -0.383)	-0.622 mV	(1.973, 0.729)	**0.091**	N/A	21.008
	FTI	-1.994 mV/s	(-2.394. -1.593)	-3.448 mV	(-5.019, -1.877)	1.584	21.008	N/A
+ At least 10 prep & no prep trials each (n=11/24)	Prep.	-0.582 mV/s	(-0.992, -0.172)	-0.461 mV	(-2.087, 1.166)	N/A	**0.105**	0.911
	No Prep.	-0.046 mV/s	(-0.395, 0.302)	-0.335 mV	(-1.868, 1.198)	**0.105**	N/A	3.719
	FTI	-1.125 mV/s	(-1.716, -0.535)	-3.549 mV	(-5.550, -1.548)	0.911	3.719	N/A
+ Frequency of being probed between 30% and 70% of trials (n=12/24)	Prep.	-0.663 mV/s	(-1.136, -0.191)	-0.430 mV	(-2.122, 1.262)	N/A	**0.108**	11.706
	No Prep.	-0.327 mV/s	(-0.665, 0.010)	-0.958 mV	(-2.387, 0.470)	**0.108**	N/A	14.823
	FTI	-2.054 mV/s	(-2.511, -1.597)	-3.263 mV	(-4.929, -1.597)	11.706	14.823	N/A

Supplementary Table 1: Summary of estimated pre-probe EEG slope [-1s, 0s] and amplitudes [-0.25 s, 0s] (times relative to probe onset), each with their confidence intervals, for Prep, No Prep, and FTI conditions under different participant retention criteria. Also given are Bayes factors (BF_{10}; Bayesian ANOVA in JASP), comparing RP amplitude in [-0.25s, 0s] across conditions. For all the retention criteria that we tested, when comparing Prep vs No Prep (bolded), we found Bayes factors around or below 0.1. This provides moderate to strong evidence that the EEG signals in the two conditions are the same, regardless of the retention criteria we used.

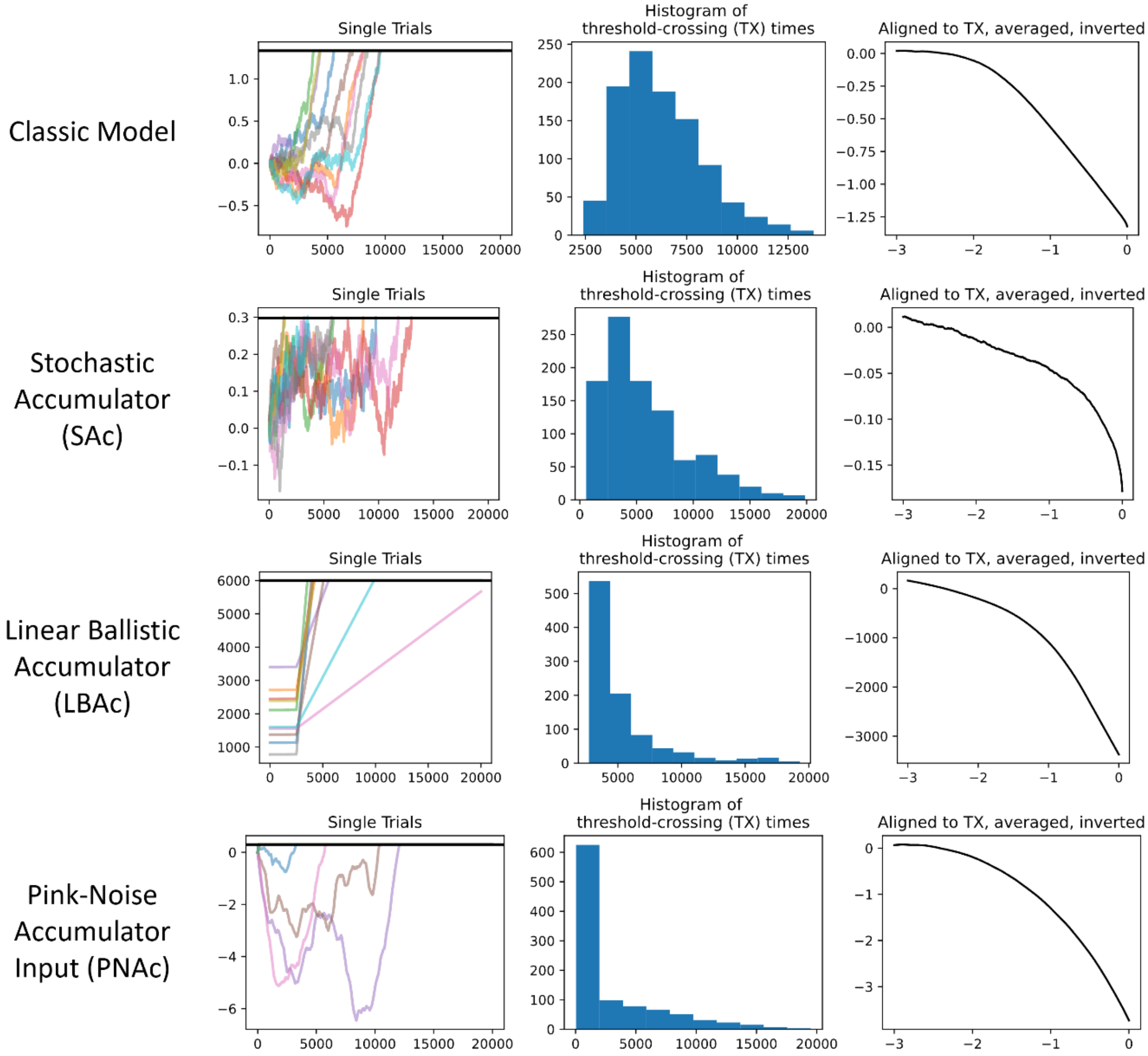

Supplementary Figure 2: Some individual runs (left), the threshold-crossing distribution (middle), and threshold-aligned back-averages (right) of four different accumulator models. Classic model: noisy fluctuations occur until an ‘unconscious decision’ is made, after which a strong input drives activity towards the threshold. Stochastic accumulator (SAc): noisy fluctuations accumulate and trigger movement after crossing a threshold. Linear ballistic accumulator (LBAc): after 2500 ms, a linear drive (drawn from a normal distribution, in addition to a starting value) pushes activity and triggers movement after crossing a threshold. Pink-noise accumulator (PNAc): autocorrelated noise (1/f exponent = 1.5) accumulates and triggers movement when crossing a threshold. All four model types recreated a long-tail distribution of threshold-crossing times (or wait times), and all recreated an early deflection as seen in the RP. However, only SAc with dual-stage metacognition explained our EEG results (Fig. 4).

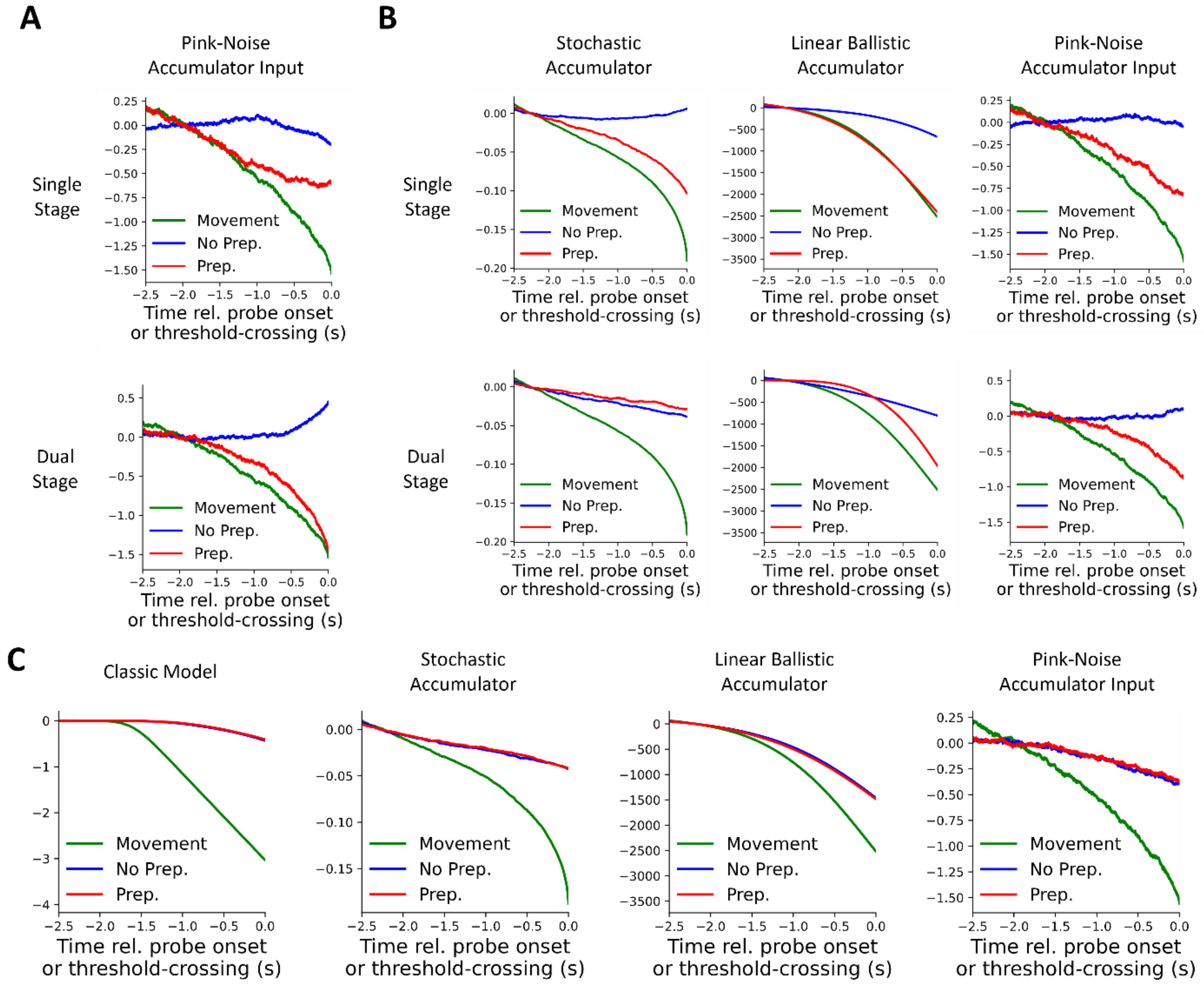

Supplementary Figure 3: Supplementary modeling results. A. Pink-noise accumulator model(Schurger, 2018). In this model, the RP reflects an autocorrelated noisy input to a drift-diffusion process rather than the process itself. Aligned to threshold-crossings, the noisy input will also show an early negative deflection akin to the RP. As in Figure 4C, we show modeling results where the decision to report ‘Yes’ or ‘No’ in response to the probe is based on the amplitude (single-stage) or slope (dual-stage) of the accumulator (not on the noisy input) at or immediately after the time of the probe. Like our findings for the stochastic accumulator model, dual-stage metacognition with this process can explain our ERP results. Furthermore, we did not find a difference between RPs on trials where participants waited a short versus long amount of time to move (Fig. S1B), which was the crucial difference that used to support this model over the standard stochastic accumulator model(Schurger, 2018). **B.** Model results if the data were split according to accumulator amplitude (single-stage) or slope (dual-stage) 400 milliseconds following probe onset, rather than immediately following the probe. This would presumably be the case if some time was required for auditory processing and task-shifting. Though not identical, the results are largely the same with those obtained from the model without a delay (compare Fig. 4C). **C.** Model results if participants’ reports about awareness of preparation are based on unrelated brain activity *or* are pure guesses. We conducted similar analyses to Figure 4, except non-movement and

non-FTI trials were randomly designated as Prep or No Prep, as one would expect if intention awareness was independent of the process that generates the RP or if reports were mere guesses. All four models were able to recreate our ERP results under these conditions (no difference between Prep and No Prep, and a negative slope before probe onset in both of those conditions). However, random guesses are unlikely because our participants reported being unsure on a relatively small percentage of trials (~9%, Fig. 2). Furthermore, the probability of reporting that they were preparing to move increased with trial duration ($p = 0.001$; see behavioral results). We therefore think the most likely case is that participants metacognitively access stochastic accumulation following probe onset (Fig. 4) or base their reports on another feature of neural activity that is minimally dependent on the RP (e.g. beta power, Fig. 5). Nevertheless, these results clarify that a classic or linear ballistic model underlying the RP is mutually exclusive with reports of motor preparation being related to the RP at all—one cannot have it both ways.
